# A Smart Congestion Control Mechanism for the Green IoT Sensor-Enabled Information-Centric Networking

**DOI:** 10.3390/s18092889

**Published:** 2018-08-31

**Authors:** Rungrot Sukjaimuk, Quang Ngoc Nguyen, Takuro Sato

**Affiliations:** Department of Communications and Computer Engineering, Faculty of Science and Engineering, Waseda University, Shinjuku-ku, Tokyo 169-0051, Japan; quang.nguyen@aoni.waseda.jp (Q.N.N.); t-sato@waseda.jp (T.S.)

**Keywords:** Information-Centric Networking (ICN), Future Internet (FI), Internet of Things (IoT), Wireless Sensor Networking (WSN), green networking, next generation communications

## Abstract

Information-Centric Networking (ICN) is a new Internet architecture design, which is considered as the global-scale Future Internet (FI) paradigm. Though ICN offers considerable benefits over the existing IP-based Internet architecture, its practical deployment in real life still has many challenges, especially in the case of high congestion and limited power in a sensor enabled-network for the Internet of Things (IoT) era. In this paper, we propose a smart congestion control mechanism to diminish the network congestion rate, reduce sensor power consumptions, and enhance the network performance of ICN at the same time to realize a complete green and efficient ICN-based sensor networking model. The proposed network system uses the chunk-by-chunk aggregated packets according to the content popularity to diminish the number of exchanged packets needed for data transmission. We also design the sensor power-based cache management strategy, and an adaptive Markov-based sensor scheduling policy with selective sensing algorithm to further maximize power savings for the sensors. The evaluation results using ndnSIM (a widely-used ICN simulator) show that the proposed model can provide higher network performance efficiency with lower energy consumption for the future Internet by achieving higher throughput with higher cache hit rate and lower Interest packet drop rate as we increase the number of IoT sensors in ICN.

## 1. Introduction

Nowadays, communication represents one of the most important parts of our interconnected world because the Internet is a fundamental technology that provides many beneficial applications for our society and daily lives. From the advantage of the Internet architecture, Internet of Things (IoT) has been developed in which different types of devices or objects things can make decisions, communicate, and exchange data with each other [1]. Thanks to the simple deployment and various practical applications, wireless sensor networks (WSNs) [2] have become a critical factor for the Future Internet (FI) paradigm in the IoT era with the rapidly growing number of Internet users. Among various proposed FI architectures, Information-Centric Networking (ICN) [3] is a promising design as it is usually realized through the overlay approach. Typically, in ICN, additional network components are added to perform the functions of data naming, and content caching by establishing data flows between content locations and content consumers for the goal of matching the desired content for user requests [4]. However, a huge number of the request packets is generated in ICN due to its packet flooding strategy, which causes high traffic, then increases the congestion rate [5] and network devices power consumption as analyzed in our previous work [6,7,8]. The energy efficiency issue even gets more challenging in the sensor-enabled network because energy consumption is a critical key in the design of WSNs as sensors are small devices with power-constraint due to their limited battery capacity.

To address the ICN congestion issue in sensor networking, in our previous work [9], we proposed a dynamic congestion control mechanism for Named Data Networking (NDN) [3], a commonly used ICN platform. The proposed network system transmits the content with content popularity and priority-based delay time, together with adaptive content lifetime, and cache management strategy. We investigated that the proposed model can provide higher network performance efficiency for the future Internet when we increase the number of IoT sensors in ICN. However, in that study, we only considered reducing the network congestion rate without resolving the network power consumption problem, one of the critical keys in a sensor-enabled network, to realize a sustainable green communication for future networks.

In this research, we propose a smart congestion control mechanism for ICN-based wireless sensor networks in the context of IoT, given that a sensor network consists a large number of small battery-powered devices which monitor, record, analyze, and process the surrounding environment wireless information for various practical applications [10,11,12]. Particularly, in our ICN wireless sensor-enabled network scenario, the proposed ICN model utilizes wireless sensors as content producers to send data with attached content popularity, together with chunk-based aggregated packets as forwarding scheme and the sensor power-based cache management policy. To minimize the sensor power consumption, the system also checks the sensors’ status using a Markov-based adaptive sensor scheduling strategy and performs suitable actions: from the set of non-inactive sensors, the server sends content requests to the appropriate sensors, and sensors then respond by replying corresponding data to the server. The proposal has four contributions as follows:

1. We utilize Markov theory to classify the sensor status into four different operating modes: active, inactive, unicast, and broadcast for the proposed adaptive sensor scheduling strategy. This mechanism can identify the optimized power profile of each state to enable maximum power saving, especially in the case of inactive mode. Particularly, we apply our sensor scheduling scheme over a proposed ICN scenario in which the system only generates content requests (Interest packets) to the sensors that are not inactive state;
2. We design a sensor power-based caching mechanism to store the content data from sensors which have their energy value less than the threshold power level value so that those content items can be served by network nodes instead of sending requests to the low-power sensors. In this way, the congestion rate in sensor-enabled ICN system is also considerably diminished due to the reduced number of Interest packets;
3. As forwarding scheme, we use a popularity-based chunk aggregation mechanism to calculate the appropriate number of chunks corresponding to the content popularity class and send these content chunks as an aggregated packet to the edge routers (i.e., routers connect to sensors). Together with the proposed caching mechanism, this forwarding scheme aims to minimize the number of the Interest packets needed for data transmission, then reduce the congestion rate in ICN substantially;
4. To resolve the energy efficiency (EE) problem in the sensor-enabled network, we use Markov-based adaptive sensor scheduling and selective sensing to maximize sensor power savings.

In short, this paper contributes to the new concept of green networking and congestion control in ICN with innovative ideas of selective sensing mechanism together with customized sensor operating modes according to the energy level of the sensors. This concept is supported by an implementation of a smart sensor power-based caching mechanism and packet aggregation method corresponding to the popularity of the content to prolong the lifetime of sensors and enable the network stability. The reason is that a request can be satisfied by a content node thanks to the proposed caching scheme or an active sensor with respective data while a content producer power is low. Specifically, by supporting multiple available sensors as content producers to serve a content, the system can redirect packet to more capable sensors and content nodes in ICN interconnections to avoid unnecessary communications via constrained producers for the overall significant EE performance improvement over the sensor-enabled ICN system. The proposal with related combined technique is therefore practical and feasible from both research and application perspectives by tackling the EE issue in a sensor-enabled network towards efficient communications in IoT era, given that a sensor has resource constraints, in terms of memory, processing ability, and power.

To the best of our knowledge, this proposal is the first work which integrates both green networking and efficient power-based caching scheme as well as content chunk aggregation as a potential hybrid wireless sensor-enabled network access solution which uses practical caching and forwarding schemes to leverage the sensor power saving for efficient potential content-based services in ICN. This proposal then realizes a practical and efficient Green networking approach in ICN architecture corresponding to the sensor status and content popularity to enhance the ICN migration process towards various dynamic content-oriented Next-Generation wireless applications in the Big Data era.

The rest of the paper is organized as follows: in Section 2, we describe related work, then the network topology, and the proposed schemes are elaborated in Section 3. We present the operating strategy for greening the sensor-enabled network in Section 4, and the energy models for EE analysis are shown in Section 5. We then analyze and discuss the evaluation results in Section 6. Finally, in Section 7, we present a summary of this study and conclude the paper with future work.

## 2. Related Work

In this section, we present the literature review related to our proposal for the goal of realizing a complete green and efficient ICN-based sensor networking framework with minimized network traffic rate and low energy consumption at the same time.

### 2.1. Information-Centric Networking (ICN)

ICN is a promising FI design, which focuses on in-network caching and named content for efficient content dissemination. Though several Internet architectures have been proposed for the FI, ICN has been considered as the global-scale FI paradigm, thanks to its benefits over existing IP-based Internet architectures. Typically, ICN can improve major network metrics, e.g., data rate, network utilization, and especially latency, compared to the current host-centric Internet because different from the existing host-to-host Internet design, the requested content data can be served by a replica from a content node in ICN interconnections, instead of only from the content source. The main concepts of ICN are named data, in-network caching, and multicasting. These three ICN fundamentals allow network elements to be aware of the content requests then aggregate multiple requests of the same content for optimizing bandwidth usage [13]. Also, many caching strategies can be utilized to improve the overall ICN performance efficiently, e.g., the policy for ICN caching and routing as defined in [14].

Although naming, caching, and forwarding mechanisms are among the core parts of ICN, which lead the direction of the ICN research, congestion control also takes a critical part as a huge number of content requests from users due to ICN request packet flooding mechanism can make the network congested, especially in the case of sensor networking in the IoT era. The reason is that all sensors in a sensor-enabled network are connected to the Internet so that all the collected data can be transmitted to server/data center for analysis and used in real-life applications [5,9]. This mechanism then produces high congestion rate due to the high workload for communications between sensors and server. Also, congestion is significant for practical ICN deployment. The reason is that the content nodes with higher centrality degree will have to work much more frequently compared to other nodes. This tendency causes a high congestion rate because of the imbalanced network operation status. Along this line, congestion avoidance and congestion control mechanisms are two major approaches to handle congestion problems. Congestion avoidance mechanisms aim to prevent the network from becoming congested whereas congestion control’s objective is to recover the network from the congested state, respectively [15].

To reduce the congestion rate of ICN systems network resource management is among the most efficient policies as cache hit rate is the primary performance metric to evaluate ICN efficiency. For this, several cache management schemes have been studied by recent research papers. For instance, [16] proposed a cache aware traffic shaping policy based on content popularity to assign the appropriate bit rate for video transmission. Recently, the authors in [17] proposed a hop-by-hop congestion control mechanism by defining the max/min threshold of Interest rate to prevent the network from congestion between the consumer and the routers. In [18] the authors presented a congestion-aware cache policy to determine which content should be cached or evicted. Also, ref. [19] proposed a utility-driven cache partitioning scheme for allocating cache partitions with different sizes.

Moreover, the in-network caching capability in ICN also raises energy consumption problems due to additional energy for caching capability compared to an IP-based network system, as analyzed in our previous work [6,7,8]. Also, although energy consumption is a major concern in the design of the future wireless communications [8], there is not much work about EE in ICN, especially in the wireless environment. In fact, most of the existing wireless ICN researches tackled with content consumers and publishers mobility to verify the benefit of ICN over IP in wireless environment. For instance, the authors in [20] proposed an AI-based approach utilizing reinforcement learning as an intelligent content prefix classification scheme for optimizing Quality of Service (QoS) in wireless ICN environment. The EE issue in ICN then raises the need for an efficient wireless green ICN-based platform for future communications with various practical applications, given the rapid increase in energy price, number of wireless/mobile content users, and huge content demands in the future network.

### 2.2. Energy-Efficiency (EE) in ICN and Research Motivation for the Green IoT Sensor-Enabled ICN System

For green networking, a combination of rate-adaptivity and power-aware routing for greening approaches was stated in [21] to save network power consumption by giving a simple model of power based on two kinds of link states (on and off), with a comparison on the benefit of each one. As the power consumption of the network system can be taken as a function of network devices status, sleep scheduling algorithm was proposed in [22] as an efficient method to save device energy. Recently, the authors in [23] studied an overview of green IoT and its challenges due to the energy usage of IoT devices. They also proposed a potential strategy that can be used to minimize the energy consumption in IoT. Also, the researchers in [24] proposed the developments and future vision of sensor-based cloud, which is a novel paradigm in green IoT for connected smart cloud services. In [25], a novel energy efficient and context-centric framework for the IoT addressed data handling and processing functions to optimize the energy efficiency of the system while effectively handling the heterogeneous QoS requirements of the applications.

For EE formulation and evaluation, an energy model is the key to evaluate the EE performance of a networking model. The authors in [26] proposed energy minimization by utilizing the three-parameters generalized inverse Gaussian (GIG) family, in which the GIG class subsumes many key two-parameters first passage time distribution families including the Gamma and IG distributions. In [27], the authors studied the energy prediction using a Gaussian process egression algorithm. Also, the authors in [28], proposed the information capacity of the GIG neuron model as an expended function of energy.

To model different state transitions using Markov-based finite state machine, recently, the authors in [29] mention that there is a lack of theoretical models that can predict future power consumption and residual availability of energy in a sensor node. They proposed Multiple boArd marKov model for Energy haRvesting Sensors (MAKERS), a Markov model-based method to capture the energy states of such sensors. This method predicts the probability that a node becomes failure due to lack of energy. The authors in [30] formulated the data recovery problem in the wireless sensor networks which is based on the Markov Random Field (MRF). Particularly, they proposed an Energy Minimization Data Recovery Algorithm (EMDRA), for the correlations among the sensory data using the spatial and temporal characteristic of the physical environment.

Regarding green networking in ICN environment, in our prior work, we applied Adaptive Link Rate (ALR), one of the well-known green networking techniques, to reduce the power consumption of network systems in ICN by dynamically varying link rate to the optimal utilization, which matches the requested content popularity quickly [6,7,8]. In addition, we also dealt with both cost-efficiency (low energy consumption) and effectiveness perspective in the concept of wireless communication: In [31], we integrated proactive-caching based scheme and smart scheduler in our proposed green ICN model for efficient communications in Intelligence Transport System (ITS) to address and solve the key problem spaces of ICN, namely: scalable, cost-efficient content distribution, mobility and disruption tolerance of ICN for FI. Also, the formulated in-network caching with a realistic energy consumption model for an ICN router as an optimization problem was applied in [21] to minimize the entire network energy.

Moreover, in ICN as multiple content request and data can result in significant delays, high collisions, and packet loss rate, Cheng et al. [32] proposed an adaptive forwarding scheme as an efficient congestion control mechanism in NDN. Also, several congestion control mechanisms have been proposed for ICN such as Receiver-driven TCP-Reno congestion control and Multi-thread congestion protocol [33], and data-aggregation techniques in sensor network as surveyed in [34,35,36]. Towards this end, we apply the concept of sensor scheduling strategy and perform efficient power-aware caching and forwarding schemes for the IoT sensor with simple and self-scalable domains for various wireless applications in ICN. Thus, the combined approach acts as an energy-aware communication design to solve the EE issue for future communications, especially in the context of the IoT sensor-enabled networking. Regarding ICN deployment, in this research, we select NDN as the ICN prototype because it is designed for the goal of network scalability, security, robustness, and efficiency by utilizing in-network caching and content naming scheme.

## 3. Proposed Smart Congestion Control Mechanism for the Green IoT Sensor-Enabled Communications in ICN

This section states the proposed communication model in a wireless environment to yield the efficient congestion control mechanism with high energy savings in IoT sensor-enabled ICN.

### 3.1. Communication Topology and Assumptions for the Green IoT Sensor Communications in ICN

**Figure 1:**
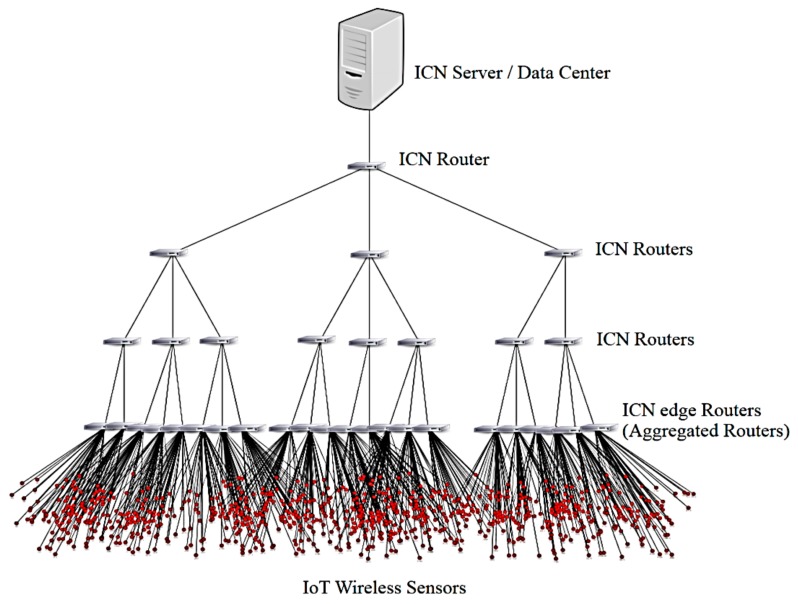
The proposed IoT sensor enabled Information Centric Networking Topology.

We select the tree topology to closely reflect how the content data varies at different levels of the network topology (Figure 1). This type of topology also represents typical sensor-enabled content dissemination scenarios for various practical wireless applications. In this model, a wireless sensor connects to the ICN edge routers, and we assume that each ICN Router can store content data in its cache storage and has all the features as of an NDN node. Particularly, the forwarding scheme of NDN includes three fundamental components, namely Forwarding Content Store (CS), Information Base (FIB), and Pending Interest Table (PIT) [37]. In NDN, consumers request their desired data by sending Interest packets (content requests) to the network and producers will reply the corresponding Data packets of the desired content. Also, NDN features support application-driven approaches for sensor networking in practice.

In this network system model, as the sensors are used to collect, store and analyze different data, the system checks the sensors’ status using adaptive sensor scheduling policy and performs suitable actions: the server only sends content requests to the appropriate sensors which are not in the low power mode and have the requested content data. The sensors then response the content request by replying corresponding data to the server.

This research focuses on IoT sensor-enabled scenario in which sensors are utilized to sense and analyze the surrounding data. Hence, we assume that a wireless sensor device only moves within a specified area managed by an edge router as a Point of Attachment (PoA), given that all ICN edge routers (Wi-Fi access points) have the same transmission radius. We define the core ICN router is at level 1 of the topology and it is connected to the data server (root node). Also note that the proposed system is not limited in the scope of the simple tree topology as we can apply the proposal to various dynamic hierarchical network models where the wireless edge routers (PoAs) connect directly to the sensors, and link to other ICN intermediate routers at upper level of network topology simultaneously.

### 3.2. The Integration of Green Networking into the Proposed Model

This section discusses a smart sensor scheduling scheme with selective sensing strategy and customized sensor energy policies to enable optimized operating nodes for maximizing the power saving gains.

#### 3.2.1. Adaptive Sensor Scheduling Policies with Customized Sensor Operating Modes

For the adaptive sensor scheduling, we adopted Markov model [30] to classify the sensor operation modes into four different statuses as: active, broadcast, unicast, and inactive, respectively (detail of Markov-based mechanism will be clarified in Section 4). Typically, in our scheme, for simplicity, each sensor operating mode has different energy consumption level in which only the active mode consumes full power capacity, and broadcasting mode consumes higher power than unicast mode whereas the inactive mode saves power the most. To implement sensor scheduling with the optimized sensor operating mode, firstly the system identifies the current sensor energy level and capability of each sensor, then adapts its operating mode to the power of the matched operating profile to save sensor energy. Also, to reduce Interest traffic due to flooding mechanism in ICN for further optimizing network system power saving gain, the server must stop sending interest requests to the sensors whenever the system detects that they are in inactive mode state. Hence, our proposed green networking design can provide the higher network efficiency in ICN in terms of both EE and network traffic saving, especially in the context of Green IoT sensor networking.

**Figure 2:**
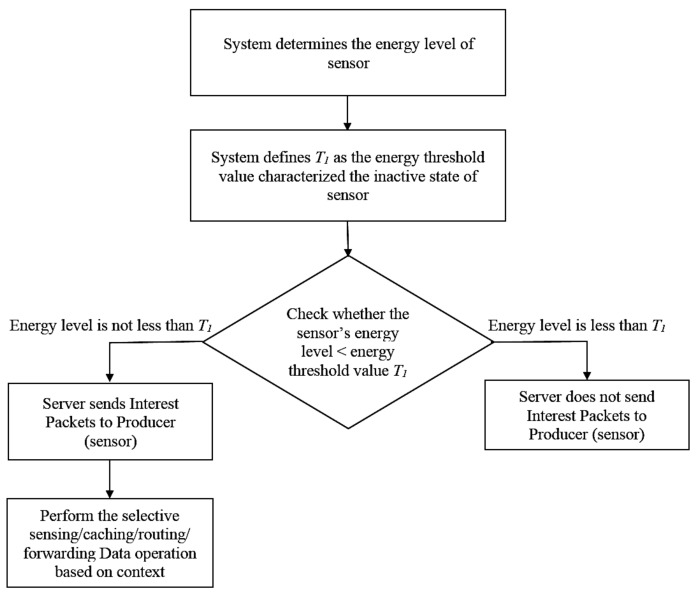
The proposed adaptive sensor scheduling flowchart.

As shown in Figure 2, to decrease the congestion rate in ICN, we propose an adaptive sensor scheduling scheme for minimizing the number of transmitted interest packets. Specifically, we characterize *T*_1_ as the threshold value of the sensor’s inactive state. If the system identifies that the sensor energy level is less than *T*_1_ (i.e., the sensor in inactive mode), the server does not send Interest packets to the sensor to minimize the congestion rate due to packet flooding mechanism in ICN. Otherwise, the server sends interest packets to the sensor and performs the selective sensing, caching, routing and forwarding data operation based on context. This mechanism will be stated in the next sections.

#### 3.2.2. Selective Sensing Mechanism

**Figure 3:**
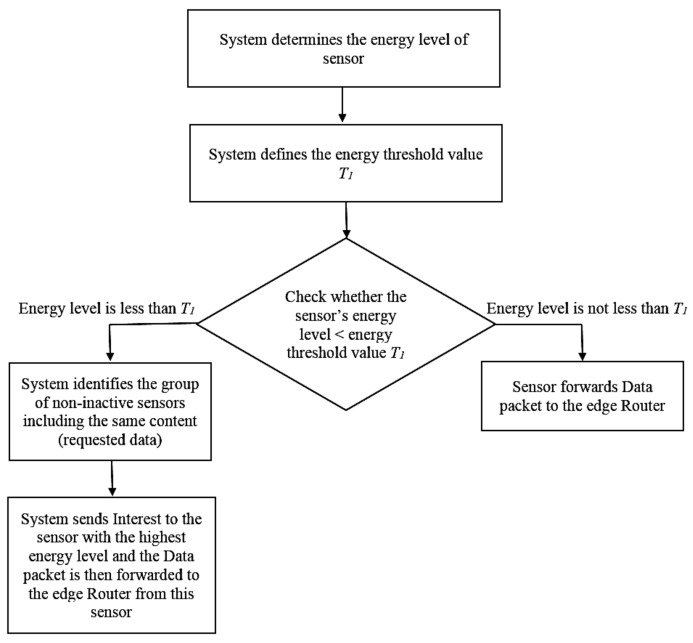
The proposed selective sensing for power control mechanism.

Regarding selective sensing for power control, when the system identifies that a sensor’s power level is less than the threshold value *T*_1_, the inactive mode is activated for the sensors to maximize the sensor’s power saving. Otherwise, the sensor then performs sensing, routing or forwarding content to intermediate ICN routers as shown in Figure 3.

In particular, in the situation when a sensor is in the inactive mode and the server needs to get data content from it, we consider that the requested data should be served by another sensor with the matched content from the network. We then propose selective sensing mechanism to improve the QoS in ICN (Figure 3). To do this, the system firstly determines the current sensor energy level then compares that value with *T*_1_. If the sensor energy level not less than *T*_1_ value, then the system forwards the content to the edge router. Otherwise, the system defines the group of non-inactive sensors including the same desired content data. Then, the system selects sending the Interest packet to the sensor with the highest energy level and the data will be forwarded to the attached edge Router from this sensor.

### 3.3. The Proposed IoT Sensor Congestion Control Scheme for Efficient Communications in ICN

In this study, we propose a smart congestion control for the green IoT sensor enabled ICN, in which the content producers (sensors) receive the request packets from the ICN server, then send content data with its attached content popularity. We integrate cache management policy and chunk-based aggregated packets for forwarding scheme to realize an efficient ICN communication model with low congestion rate gained from the reduced network traffic.

#### 3.3.1. Aggregated Popularity-Based Forwarding Scheme with Chunk-by-Chunk Data Packets

In this section, we discuss a smart forwarding scheme, under the assumption that a chunk is the unit for data transmission [3]. We then design a chunk-based aggregation forwarding mechanism to reduce network traffic to further enable network system resources savings. Through the observation that content objects would be found in the caches of ICN router nodes, we adopt the aggregated chunk-based forwarding scheme (according to the popularity levels of content items) to reduce congestion rate of a content node (CN).

To characterize the content priority and content popularity levels, we take Zipf distribution-based model [38], and its formula can be defined as follows:

(1) $$f\;\left(k\right)=\frac{k^{-\alpha }}{\sum_{i=1}^{F}i^{-\alpha }}\;,$$

where *k* is a rank of the content, *α* is the skewness factor characterizing the Zipf distribution, and *F* is the total number of content items (files). From the return value of *f* as defined in (1), we can identify the content popularity level of a specific content.

When the content is transmitted through the intermediate routers (from the IoT sensors as the content sources), the system assigns an appropriate number of chunks for data aggregation before forwarding the content to the intermediate routers. In particular, this number is based on content popularity and the content chunks will be aggregated at the sensor in order of chunk number, i.e., start from the foremost chunks, then forwarded to the edge Router.

To do this, let *K_pop_* be the rank of content popularity, then each sensor’s data aggregation method is identified corresponding to the *K_pop_* value of the content that the sensor serves. Particularly, each sensor employs a chunk-based aggregated number characterized by a *K_pop_* value of the content. Based on this value, an adaptive chunk-level forwarding policy will be applied for each arriving content to the attached edge router. Let *Chunk_ratio_* be the chunk-by-chunk aggregated-ratio of the content item (start from foremost chunk) and *Chun_kall_* be the total number of chunks of a content. Then, the proposed forwarding aggregated ratio for a specific content from a producer (sensor) can be identified by the following formula:

(2) $$Chunk_{ratio,}=\left[\;\frac{K_{low}}{K_{pop}}\;\right]\;Chunk_{all\;}$$

where *K_low_* indicates the rank of lowest content among the most popular content class.

In this way, only the most popular content items are fully-transmitted to the edge router. All the remaining content are then only partially-forwarded to the aggregated router for distribution in ICN interconnections via the in-network caching mechanism.

**Figure 4:**
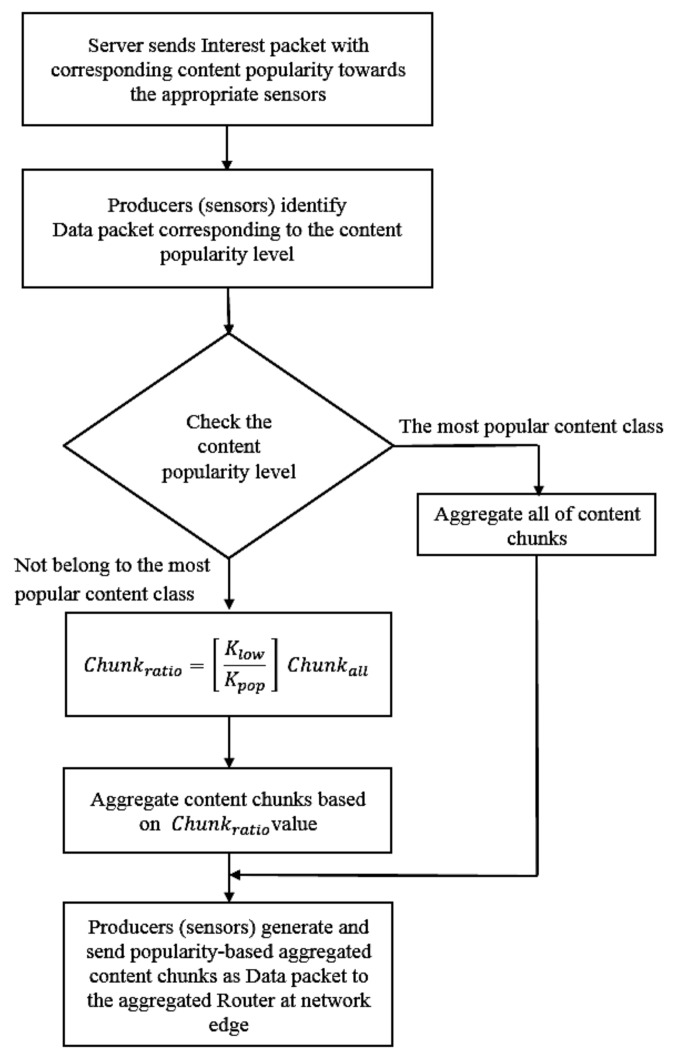
The proposed chunk-based aggregation flowchart.

The detailed chunk-based aggregation scheme is depicted in Figure 4, in which the server sends Interest packets with the corresponding content popularity to the appropriate sensor with matched data. Next, the sensors generate the data packets by aggregating content chunks based on popularity of the content. Particularly, to reduce traffic needs for saving network system resources, we introduce chunk-by-chunk aggregation corresponding to the content popularity. To do this, the system checks content popularity to identify whether a content is popular or not. Then, if the content is belonging to the most popularity class, all the content chunk will be aggregated as a whole for the data packet. Otherwise, the sensors send aggregated content chunks to the aggregated router at network edge with *Chunk_ratio_* according to content popularity level as defined by (2).

#### 3.3.2. Sensor Power-Based Caching Mechanism

**Figure 5:**
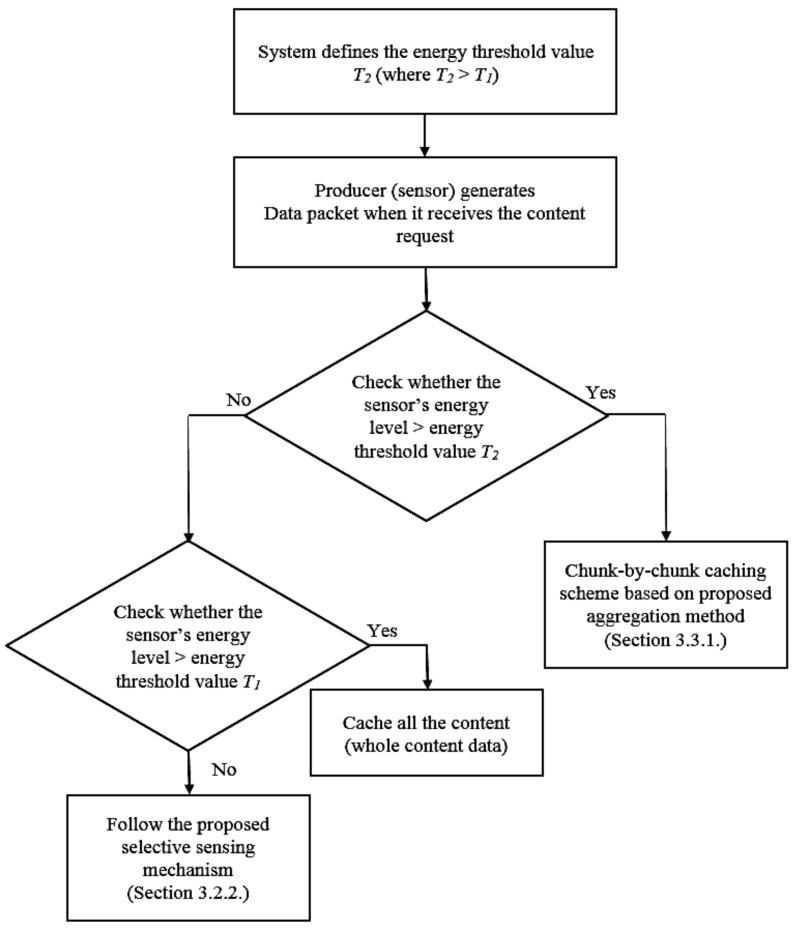
The proposed sensor power-based caching algorithm.

In this paper, we propose a sensor power-based smart caching model to increase the cache hit rate in ICN and sensor-power saving at the same time. We assume that IoT sensors are utilized for congestion control application, and when the sensors receive the content requests, they respond the data packet of the requested content, together with its power level. Then the system checks the sensor’s energy value and performs a context-based caching scheme. We denote that *T*_2_ as the threshold value of the sensor energy level with low power (where *T*_2_ > *T*_1_). In detail, a sensor with energy level *e_p_*: *T*_1_ < *e_p_* < *T*_2_ means that the sensor is limited access due to low power though it is not in an inactive mode. To realize a complete and efficient content data allocation among ICN caches, we then propose a sensor power-based caching mechanism, which can reduce the number of chunks for caching at ICN router based on its attached sensor’s power level. Particularly, the system uses *T*_2_ as the energy threshold value characterized the state of the sensor (where *T*_2_ *> T*_1_) to identify the suitable caching policy: If the sensor energy level is greater than *T*_2_ value, the content data will be forwarded and cached at the attached edge router based on our proposed aggregation method (as stated in Section 3.3.1). When the sensor energy level is higher than the threshold value *T*_1_ but less than that of *T*_2_, the sensor will send data content to its attached edge router and inform the router to cache the whole data. In this way, data from all the sensors with low power can be served by other content nodes via in-network caching mechanism in ICN to further increase the power saving in ICN. Otherwise, i.e., when the sensor energy is less than *T*_1_, the system performs the same way as the selective sensing mechanism defined in Section 3.2.2. The detailed sensor power-based caching algorithm is summarized in Figure 5.

## 4. Markov-Based Scheduling Operating Strategy for Greening ICN-Based IoT Sensor Networking

In this section, we adopt the concept of MRF [30] for modeling the transition state of sensors as the IoT devices with multiple associated power states towards the next-generation communications.

Let *X* = {*X*_1_, …, *X_n_*} is a group of random variables *X_i_*, in which *x_i_ D* and *D* is a discrete set or continuous interval. Let *P*(*X_i_ = x_i_*) be the possibility that *X_i_ = x_i_*. Then, the probability *P*(*X_i_ = x_i_*) and the joint probability *P*(*X_i_ = x_i_*, *…*, *X_n_ = x_n_*) then can be unified as *P*(*x*). The condition that *X* is the MRF of a sensor *S* in the set of a sensor’s all available states *N* can be described as:

(3) P(x),>0, ∀ x ∈ X

(4) $$P(x_{i}|\;x_{S-\{i\},})=P(x_{i}|\;x_{Ni})$$

in which *i* and *j* denote the current and next operating power state, respectively, *N_i_* is the set of all available upcoming states of a sensor from its current state *i*, *x_Ni_* = {*x_j_*| *j N_i_*}, and 𝕏 = *D^n^*. In case that *D* = *ℝ*, 𝕏 = *ℝ^n^* represents the real number space with *n* dimensions.

In this proposal, we utilize adaptive Markov-based smart scheduling operating strategy for Greening ICN-Based IoT Sensor Networking. We define the sensor status from four proposed sensor power operating four modes as: active, broadcast, unicast, and inactive respectively. In other words, we define one state for each sensor at a time and schedule the sensor state in the next period, with the constraint that a sensor with higher energy state can only transit to a lower energy state, not in the inversed way. Particularly, we utilize the Markov model to identify the probabilistic finite sensor state transitions in which if a power state has more than one available upcoming state (e.g., an active mode can move to all other states), the probability of the transition will be higher when the power gap of two states is small, and vice versa.

Then, to identify the possibility of the optimized upcoming sensor power status of *S* (*S_S_*), we adopt the concept of MRF, where *S_S_* can be identified as follows:

(5) $$S_{S}=P(x_{i}|\;x_{S-\{i\},})\;$$

$$p_{i}>p_{j}\;,$$

where *p_i_* and *p_j_* denote the power state of *i* and *j*, respectively. In this way, the producers (sensors) can stay in in-active mode and extend its lifetime to maximize power saving as the requested content can be served by other content nodes or producers. Note that we have the plan to extend this Markov-based model by allowing an in-active sensor to fully-recharge its power to the active mode (i.e., transit to the highest operating mode) in our upcoming paper.

Also note that the Markov-based energy state transition is implemented by the system into a sensor as a built-in function for scheduling the sensor energy state. In detail, after the sensors change their state via the proposed MRF-based dynamic scheduling strategy, the system will perform the suitable adaptive operation by sending the content requests to only capable sensors (i.e., non-inactive producers) with corresponding data. At that time, the system will check the current energy level status of each sensor obtained by the Markov-based model and compare the energy value to the threshold value *T*_1_ and *T*_2_. Accordingly, from this estimation, the sensor adapts its operation (sensing/caching/routing and forwarding policy) based on the context as defined in Section 3. Specifically, in case one or more content producers are in the inactive mode, the system performs the selective sensing and selects only the sensor with the highest energy level to maximize the power saving and keep as many available content sources in the sensor-enabled ICN system as possible simultaneously.

## 5. Energy Models for Energy Efficiency Analysis

### 5.1. Total Energy Consumption per Successfully Transferred Bit

This section states the proposed mathematical energy models for energy savings in the proposed sensor-enabled ICN system. Typically, the energy is consumed by forwarding frames per bit during the transmission process to the receiver without error [39]. In our work, we calculate the total energy consumption per successfully transmitted bit (*E_total_*) by the following formula:

(6) $$E_{total,}=\left[\;\frac{T_{c}}{T_{b}}\;\right]\;$$

where *T_c_* is the total amount of energy consumed by the sensors, and *T_b_* denotes the total amount of data successfully delivered to the server from the sensors.

### 5.2. Energy Models

In this paper, we build the energy model to evaluate the proposed green IoT sensor-enabled system in ICN in the same way as we did in our prior work [8]. Particularly, the network system power consumption is calculated by summing up the energy consumed by all network components. Also, we assume that there are two primary network elements in our proposed sensor-enabled network system in ICN: content routers (ICN routers) and sensors with four proposed Markov-based operating modes.

## 6. Performance Evaluations and Discussion

In this section, we verify the efficiency of the proposed smart congestion control for the green IoT sensor-enabled system by simulation using ndnSIM (a widely-used ICN simulator) [40], a widely-used and scalable ns3-based NDN simulator. For the evaluations, the ICN routers are connected through wired connections, whereas wireless IoT sensors connect to the network via wireless links attached to corresponding edge router to form a complete ternary tree topology, as shown in Figure 1. We installed NDN protocol stack in each ICN router so that each ICN node has the three fundamental components of CS, PIT and FIB as of an NDN node. The energy models of the network systems for EE evaluation are defined as in Section 5.

For simplicity, we assume that all the content objects share the same size of 1 MB with a chunk payload size of 1 KB to prevent fragmentation. The simulation link capacity/bandwidth is 100 Mb/s 1 Mb/s for wired and wireless connections, respectively. The key simulation parameters are presented in Table 1. Specifically, we define power consumptions of active state, broadcast state and unicast state are 100%, 75%, and 50% as of server power capacity, respectively. We simulate five scenarios with a various number of sensors ranged from 2860 to 3300 for all of the network architectures including our proposal model and other network systems.

We adopt the Zipf distribution [38] (with *α* = 1) for the content popularity distribution because it is widely-used to model the content objects request frequency in the network. We refer to related studies to collect data of key network power elements and their energy value for calculating the total energy per successfully transfer bit [39].

Then we make simulation and comparisons the performance of various wireless network system approaches including three network designs (NDN platform stands for conventional ICN design, sleep scheduling ICN [22] and the proposed ICN model) under the same network condition and parameters as described. We use the proposed chunk-based aggregated packets as the forwarding scheme and sensor power-based cache management policy for the proposed ICN system. Also, the Markov-based adaptive scheduling policies with customized sensor scheduling modes and selective sensing as defined in Section 3.2 and Section 4 to green our proposed sensor-enabled system in ICN.

Considering average power of the overall network system is total system energy (defined from energy models and formula in Section 5) over network runtime, the following network metrics are used to evaluate the efficiency of the proposed smart congestion control mechanism for green IoT sensor network model over the conventional ICN and sleep scheduling ICN, in terms of power consumption and network performance:

1. Packet drop rate
  The ratio of dropped packets over the total number of packets in ICN systems.
2. Average cache hit rate
  The ratio of hit packets over the total number of packets gained from caching mechanism in ICN systems.
3. Network throughput
  The network throughput measured in Mbps.
4. Energy consumption per successfully transmitted bit
  The total network energy consumed per successfully transfer bit, measured in watt per bit (W/bit).
5. Transfer bit rate over total energy consumption
  The total network transfer bit per total energy consumption, measured in bit per watt (bit/W).

**Figure 6:**
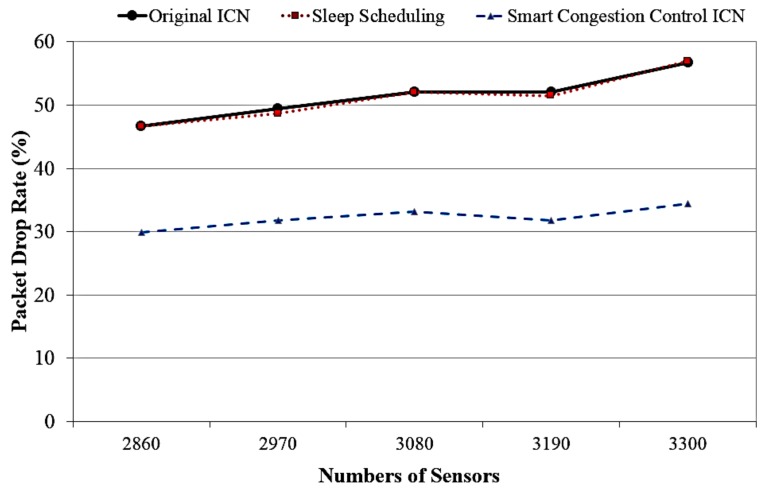
Interest packet drop rate versus different numbers of sensors.

Figure 6 depicts the Interest packet drop rate corresponding to various numbers of sensors between the proposed models, sleep scheduling ICN model, and traditional ICN design. In this evaluation, a packet drop means that an Interest packet is discarded at the ICN content node due to packet collision caused by high traffic (i.e., do not get returning data packets as acknowledgement messages (ACKs) or data packet loss after a period of time). The results show that our ICN models can get less Interest packet drop rate of content requests than other ICN systems. Especially, our proposed ICN system can get much higher performance in terms of low packet drop rate when the network scale gets larger (i.e., high number of sensors). This result confirms that the proposed system is a promising scalable solution which can gain the high network performance (low packet drop rate) for the goal of reducing the network congestion.

**Figure 7:**
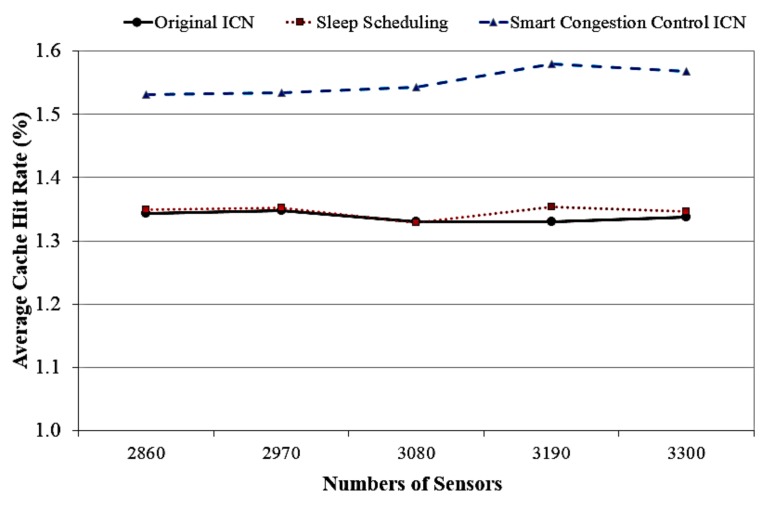
Cache hit rate corresponding to different numbers of sensors.

Subsequently, Figure 7 presents the cache hit rate of our proposed ICN model, sleep ICN model, and original ICN system in accordance with various numbers of sensors. The evaluation result shows that the proposed smart congestion control ICN can gain a higher cache hit rate than the sleep ICN model and the traditional ICN design. This is because the proposed system integrates both sensor power-based cache management scheme and chunk-level aggregation scheme for forwarding strategy.

**Figure 8:**
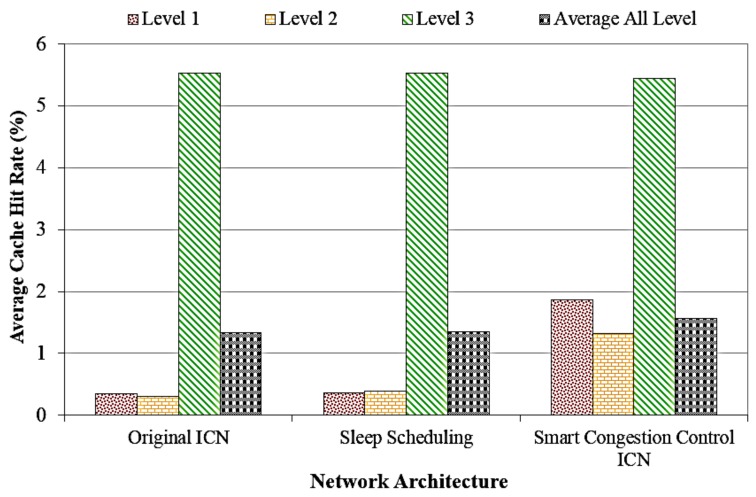
Cache hit rate versus different network levels with the highest numbers of sensors.

Following, Figure 8 analyses the cache hit rate at different network levels of the network topology of the traditional ICN, sleep scheduling ICN, and the proposed system with the highest numbers of sensors for evaluation (3300 sensors). The results show that our ICN model can provide higher cache hit rate than other ICN models, especially at the first level of network topology. Hence, consumers do not need to get the content at the producer level because many content items already stored at nearest routers which are closed to the server. Thanks to the cache management policy, the proposed system can serve a high number of content objects with much lower latency and hop-count for a higher user’s Quality of Experience (QoE).

**Figure 9:**
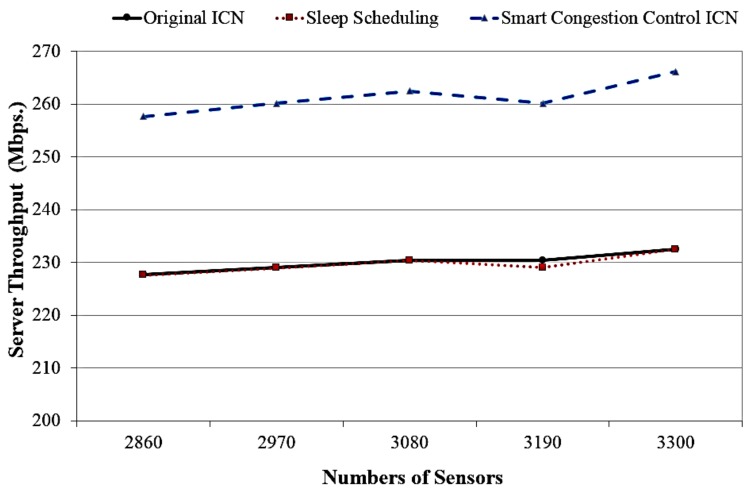
The server throughput according to different numbers of sensors.

Figure 9 represents the server throughput corresponding to various numbers of sensors of the proposed ICN model, sleep ICN model and traditional ICN. The results show that our ICN system can gain higher server throughput than other ICN design. This is because the proposed system transmits content with chunk-based aggregated packets according to content popularity value as forwarding scheme, and implements cache management scheme as defined in previous sections.

**Figure 10:**
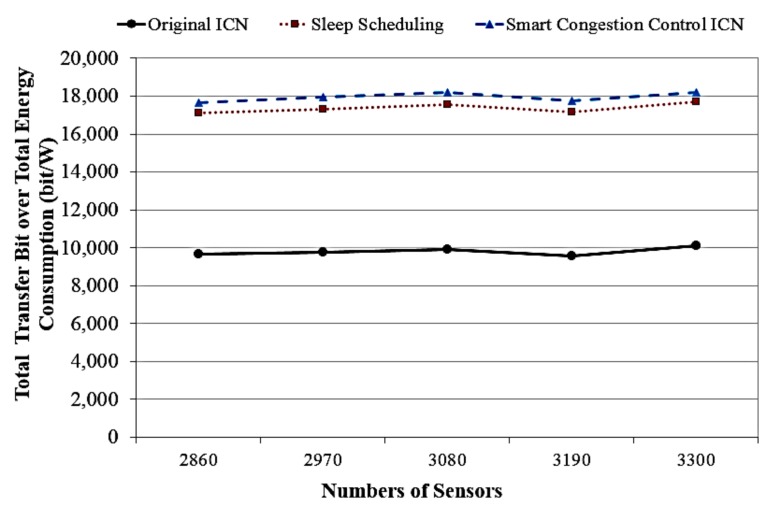
The total transfer bit versus different numbers of sensors.

Subsequently, Figure 10 shows the total transfer bit over total energy consumption versus different numbers of the different network system. The evaluation results show that our ICN system can gain higher transfer bit than the sleep scheduling ICN and the traditional ICN. This result confirms that the proposed system is a promising solution which gains the higher EE performance for the goal of enabling a cost-effective networking model utilizing sensors for the IoT era.

**Figure 11:**
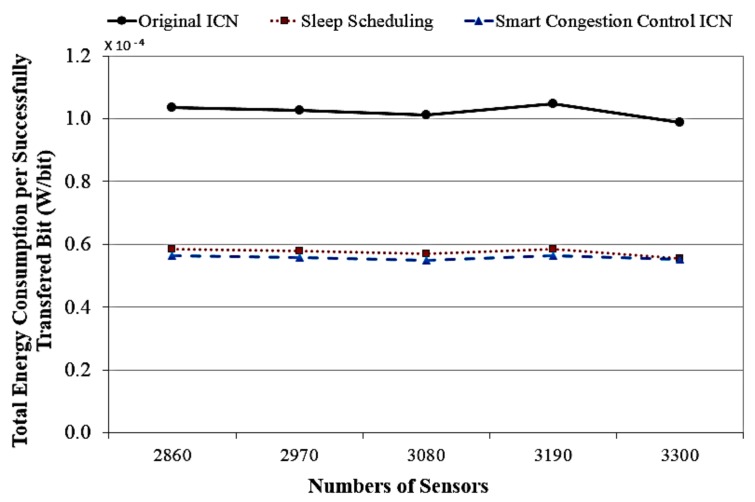
The total energy consumption of sensors accordance with different numbers of sensors.

Finally, Figure 11 illustrates the total energy consumption per successfully transmitted bit in accordance with different numbers of sensors between our proposed ICN model and the two others relevant ICN systems. A successful transmission is identified when a corresponding data packet for the requested content is not received after an Interest packet is sent. The results show that our ICN system consumed lower energy per transmitted bit than original ICN and sleep ICN system. This shows that our proposed system is an efficient solution to diminish the network congestion rate, with higher network performance and energy efficiency at the same time in the context of ICN-based green IoT sensor.

Overall, to further improve the ICN system efficiency by achieving higher network throughput and maximizing energy savings at the same time, in this study, we utilize content popularity to identify the number of chunks should be used for aggregated packets to forward data efficiently. We also proposed a sensor-power based cache management policy as well as the Markov-based adaptive scheduling strategy with selective sensing policy for greening the sensor-enabled ICN system. The evaluation results show that our smart congestion control mechanism is the efficient solution to diminish the high congestion rate with low sensor energy consumption in ICN architecture. This research is then different from our previous studies on ICN congestion control mechanism [5,9], in which we only considered reducing network traffic load for higher network performance without concerning the network power consumption problem for the practical deployment of the sensor-enabled network.

## 7. Conclusions and Future Work

In this study, we have designed a highly efficient green sensor-enabled ICN model utilizing a smart congestion control scheme for content distribution in the NDN framework. Particularly, we propose a new sensor-power based caching mechanism and Markov-based adaptive sensor scheduling policy with selective sensing policy, to solve the EE issue as well as inefficient network resource utilization of current ICN system due to the default packet-flooding policy in ICN. Also, we utilize popularity-based chunk aggregated packets for the forwarding scheme towards network caches, to reduce the number of packets transmitted to aggregated ICN routers (edge routers) and better leverage the in-network caching feature in ICN. Thanks to the smart proposed methods, this results in the network system’s reduced traffic load, and in turn energy consumption. In particular, the evaluation results show that our ICN model can provide higher network performance compared to the conventional ICN design and sleep scheduling ICN model, by achieving higher throughput and cache hit rate, with lower Interest packet drop rate and energy consumption, simultaneously. As a result, our proposed ICN can be considered as a feasible and potential solution to solve the network congestion issues in the ICN-based sensor network, especially in the context of green IoT and big data era.

Regarding future work, we will consider several content popularity models to improve the overall network efficiency under various network topologies. Regarding the aggregation method, we will design a probabilistic coefficient to aggregate content chunks of content not belong to the most popular class in which the data aggregation probability is a function of sensor residual power and its power capacity. Also, we will implement our work as a practical approach towards sensor-enabled scenario for the health-care system, which aims to get low congestion as well as high network performance in ICN for efficient health-care services.

## Figures and Tables

**Table 1:** The key simulation parameters.

Parameter	Value
Content size	1 MB
Interest request frequency/sensor	10 Interest packets/s
Payload size (chunk size)	1024 Byte
Link capacity	100 Mbps
Sensor max data rate	1 Mbps
CS size	0.01% of all available content
*T*_1_ Threshold value	25% as of sensor power capacity
*T*_2_ Threshold value Runtime	35% as of sensor power capacity 700 s
